# Highly sensitive pork meat detection using copper(ii) tetraaza complex by electrochemical biosensor†

**DOI:** 10.1039/d2ra05701h

**Published:** 2023-01-11

**Authors:** Noraisyah Abdul Kadir Jilani, Emma Izzati Zakariah, Eda Yuhana Ariffin, Suhaila Sapari, Devika Nokarajoo, Bohari Yamin, Siti Aishah Hasbullah

**Affiliations:** a Department of Chemical Sciences. Faculty Science and Technology, Universiti Kebangsaan Malaysia 43600 Bangi Selangor Darul Ehsan Malaysia aishah80@ukm.edu.my

## Abstract

Three copper(ii) tetraaza complexes [Cu(ii)LBr]Br (1a), [Cu(ii)L(CIO_4_)](CIO_4_) (2a) and [Cu(ii)L](CIO_4_)_2_ (2b), where L = 5,5,7,12,12,14-hexamethyl-1,4,8,11-tetraazacyclotetradeca-7,14-diene were prepared and confirmed by FTIR, ^1^HNMR and ^13^CNMR. The binding interaction of complex (1a, 2a, 2b) with calf thymus DNA (CT-DNA) was investigated using UV-vis absorption, luminescence titrations, viscosity measurements and molecular docking. The findings suggested that complex 1a, 2a and 2b bind to DNA by electrostatic interaction, and the strengths of the interaction were arranged according to 2b > 1a > 2a. The differences in binding strengths were certainly caused by the complexes' dissimilar charges and counter anions. Complex 2b, with the biggest binding strength towards the DNA, was further applied in developing the porcine sensor. The developed sensor exhibits a broad linear dynamic range, low detection limit, good selectivity, and reproducibility. Analysis of real samples showed that the biosensor had excellent selectivity towards the pork meat compared to chicken and beef meat.

## Abstract 2

The electrochemical biosensor of porcine using copper(ii) tetraaza complex gave excellent performance with a broad dynamic range, low detection limit, good selectivity and reproducibility. The biosensor could sensitively detect pork DNA in real meat samples.

## Graphical abstract

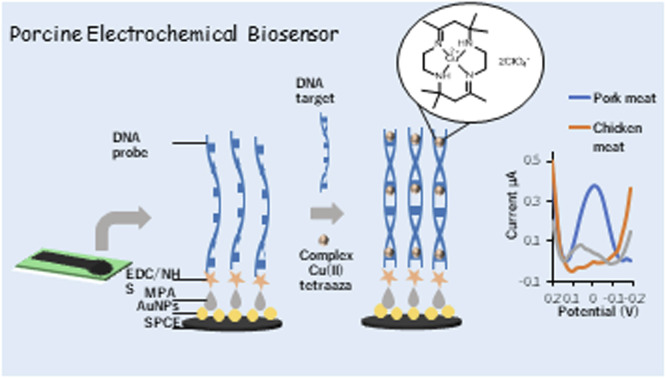

## 1. Introduction

Adulteration of pork or its derivatives in food, particularly in meatballs and sausage, continuously occurs due to its cheaper availability than other meats.^1,2^ As some religious laws do not permit consuming any food containing pork, this matter should not be taken lightly. In addition, carelessly consuming this contaminated food sometimes induces allergic effects in certain individuals. It harms people, especially those with diabetes and cardiovascular disease, since it contains high cholesterol and saturated fats.^3^ Besides, excessive intake of pork products also can lead to an increased likelihood of developing other dangerous diseases such as arthritis, osteoporosis, Alzheimer's, asthma, and infertility. Therefore, recognizing pork in foods is essential for meat authentication and satisfying market demand for protection against falsely branded foods.^4^

In this regard, a reliable approach is required for food ingredients analysis in order to identify the existence of any pork derivatives in food products. Because of this, DNA biosensors that provide simple, specific, fast, on-site analysis and easier verification is one of the suitable techniques to solve the detection of pork in food or materials. Although pork DNA electrochemical biosensors have been reported, the detection is not really sensitive.^5^

In order to improve the sensitivity, the tetraaza complexes having four amine groups were investigated. Interestingly, copper(ii) tetraaza complexes and tetraaza ligands have excellent anticancer activities.^6^ This ability is due to the presence of amine functional groups capable of becoming a host molecule that provides hydrogen bonding and can bind to biopolymers such as nucleic acid and proteins.^6^ The binding mechanism between complexes and DNA is essential for developing biosensors.

Thus, DNA interaction studies have been investigated extensively. Basically, transition metal complexes such as copper(ii) complex can typically bind to DNA through intercalation, groove binding, electrostatic and other forms of interactions.^7,8^ As cofactor agents, this complex can generate redox signals before and after the hybridization event.^9^ This characteristic allows the complex to be used as an electroactive compound to track the DNA hybridization.^10^ Previous research investigated the DNA interaction with copper(ii) macrocyclic polyamines bearing acridine moieties. The complex bind to DNA through intercalation mode due to the synthesized complex's planar moieties, which can cause it to stack between DNA base pairs.^11^ A similar mode of binding has been reported by Arthi *et al.*, where the tetraaza macrocycles with benzoyl pendant-arms with the planar environment interacted with DNA through intercalation mode.^12^ X. Li *et al.* have described a development of electrochemical detection of DNA hybridization based on copper(ii) complex *via* intercalative interaction [Cu(phen)_2_]^2+^.^13^ Meanwhile, G. Li *et al.* have reported the potential of a copper(ii) complex [Cu(dafone)_2_]^2+^ bind with double-stranded salmon sperm DNA also through intercalation mode using cyclic voltammetry.^14^ Although there are numerous DNA binding studies, none analyzed the effect of charge and size on the complex-DNA interaction, mainly on the effect of moieties.

Thus, the limitation of research on the effect of charge and size of the complex on the interaction with the DNA has instigated the synthesis, characterization and binding study of the three copper(ii) tetraaza complexes (1a, 2a, 2b). The use of copper(ii) tetraaza complex as a label for DNA detection was newly developed and showed to be an excellent electrochemical indicator. The electrochemical biosensor has been fabricated by immobilizing gold nanoparticles (AuNPs) onto the disposable screen-printed carbon electrode (SPCE), followed by the deposition of 3-mercaptopropionic acid (MPA). Activation of the layer was performed by self-assembled 1-ethyl-3-(3-dimethylaminopropyl)carbodiimide/*N*-hydroxysuccinimide (EDC/NHS), forming an intermediate ester. 2b was then applied to obtain the DNA hybridization's electrochemical response using differential pulse voltammetry (DPV). The developed biosensor has a lower detection limit and broad linear range suitable for establishing new rapid tools for porcine detection.

## 2. Experimental

### 2.1. Chemicals and instrumentations

Analytical grade quality of reagents and solvents were used without further purification. DNA was acquired from Sigma Aldrich Company. Tris(hydroxymethyl)amino methane–HCI (Tris–HCI) buffer solution was prepared using deionized distilled water. The infrared spectra were analyzed by KBr pellets and measured on a PerkinElmer 400 FT-IR spectrophotometer. Barnstead Electrothermal Melting Point IA9100 series was used to obtain the melting point. Shimadzu UV-2450 PC spectrophotometer was used to record the electronic spectra in the 200–800 nm region. PerkinElmer LS55 spectrophotometer was used to determine the emission spectra at room temperature. ^1^H NMR and ^13^C NMR spectra were obtained on a JEOL spectrometer ECP 400 MHz in d_4_-MeOH and d_6_-DMSO as a solvent. Single Crystal X-ray Diffractometer model Bruker SMART APEX CCD was used to conduct the single-crystal X-ray study. For pH titrations, a Eutech pH 700 was used to read the pH values directly. A concentrated HCI was used to adjust the acidity. Ionic strength was kept constant by adding 50 mM NaCl. The current response was measured using differential pulse voltammetry (Autolab potentiostat). SPCE used was from Scrint Technology. The oligonucleotide sequence of each DNA is as below:

Probe DNA: (5′)-CTG ATA GTA GAT TTG TGA TGA CCG TAG-(CCC-NH_2_)-(3′).

Complementary DNA: (3′)-GAC TAT CAT CTA AAC ACT ACT GGC ATC-(5′).

Chicken DNA: (3′)-GCG TCC ATA ATG ATA GTA GGT GGA G-(5′).

Beef DNA: (3′)-GTA TCG TTA ACG GTA TCA GGT GGA T-(5′).

### 2.2. Synthesis

#### 2.2.1. Synthesis of tetraaza bromide [H_2_L]Br_2_ (1) and tetraaza perchlorate [H_2_L](CIO_4_)_2_ (2) ligands, (L = 5,5,7,12,12,14-hexamethyl-1,4,8,11-tetraazacyclotetradeca-7,14-diene)

**Scheme 1:**
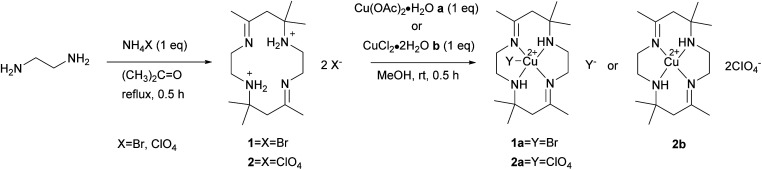
Synthesis of tetraaza ligand 1 and 2 and copper(ii) tetraaza complexes 1a, 2b and 2b.

The synthesis of the protonated ligand has been reported previously by our research group.^15^ Ammonium bromide (0.97 g, 0.01 mol) and 30 mL acetone were mixed and refluxed for 5 minutes at 80 °C. Ethylenediamine (0.60 g, 0.01 mol) was added dropwise into the solution and refluxed for 30 minutes. The solution was filtered out, and the filtrate was left at room temperature for 24 hours until a white precipitate was observed. The precipitate was washed with acetone before being dried in a desiccator. The steps were repeated using ammonium perchlorate (1.17 g, 0.01 mol) to replace ammonium bromide. The reaction for synthesizing tetraaza ligands 1 and 2 is shown in Scheme 1.

[H_2_L]Br_2_ (1) Yield: 72%; m.p. 113.4–115.3 °C; UV-vis *λ* 246 nm, *ε* 333 M^−1^ cm^−1^; FT-IR (KBr) (*ν*_max_/cm^−1^): 3460, 3402 (N–H), 2980 (C–H), 1665 (C=N), 1226 (C–N). ^1^H-NMR (d_4_-MeOH, 400 MHz): 1.49 (s, 6H, (CH_3_)_2_), 2.06 (s, 3H, CH_3_), 2.81 (s, 2H, CH_2_), 3.43 (t, 2H, CH_2_–NH_2_^+^, *J* = 4.8 Hz), 3.70 (s, 2H, CH_2_–N=C); ^13^C-NMR (d_4_-MeOH, 400 MHz): 20.4 (CH_3_)_2_, 23.3 (CH_3_), 41.2 (C(CH_3_)–CH_2_–C(CH_3_)_2_), 43.7 (CH_2_–NH_2_^+^), 46.9 (CH_2_–N=C), 58.4 (C–(CH_3_)_2_), 175.8 (C=N) ppm.

[H_2_L](CIO_4_)_2_ (2) Yield: 76%; m.p. 175.8–176.7 °C; UV-vis *λ* 238 nm, ε 403 M^−1^ cm^−1^; FT-IR (KBr) (*ν*_max_/cm^−1^): 3466, 3407 (N–H), 2981 (C–H), 1667 (C=N), 1114 (C–N). ^1^H-NMR (d_6_-DMSO, 400 MHz): 1.33 (s, 6H, (CH_3_)_2_), 1.92 (s, 3H, CH_3_), 2.65 (s, 2H, CH_2_), 3.39 (m, 2H, CH_2_–NH_2_^+^), 3.39 (m, 2H, CH_2_–N=C), 8.56 (s, 2H, NH_2_^+^); ^13^C-NMR (d_6_-DMSO, 400 MHz): 21.5 (CH_3_)_2_, 24.5 (CH_3_), 41.3 (C(CH_3_)–CH_2_–C(CH_3_)_2_), 43.6 (CH_2_–NH_2_^+^), 47.3 (CH_2_–N=C), 58.2 (C–(CH_3_)_2_), 174.7 (C=N) ppm.

#### 2.2.2. Synthesis of metal complexes

[Cu(ii)LBr]Br (1a). Complex 1a was obtained from the reaction of ligand 1 (47.8 mg, 0.1 mmol) and a metal salt, Cu(OAc)_2_·H_2_O (20 mg, 0.1 mmol), in methanol. For 30 minutes, the mixture was constantly mixed, yielding a purple solution. The solution mixture was filtered, and the filtrate was left to vaporize at room temperature. The complex of 1a was acquired as purple needles from the concentrated solution. Then, the reaction products were rinsed repeatedly with ethanol, followed by drying in a desiccator.

[Cu(ii)LBr]Br (1a): Yield: 86%; m.p. 190.3–190.8 °C; UV-vis *λ* 257 nm, *ε* 9444 M^−1^ cm^−1^; *λ* 504 nm, *ε* 194 M^−1^ cm^−1^; FT-IR (KBr) (*ν*_max_/cm^−1^): 3217 (N–H), 2968 (C–H), 1669 (C=N), 1163 (C–N). The data were in accordance with the literature.^16^

[Cu(ii)L(CIO_4_)](CIO_4_) (2a) and [Cu(ii)L](CIO_4_)_2_ (2b). Complex isomer 2a was obtained by reaction of ligand 2 (48.1 mg, 0.1 mmol) and a metal salt, Cu(OAc)_2_·H_2_O (20 mg, 0.1 mmol), in methanol. The mixture was continuously mixed for 30 minutes, producing a purple solution. The purple mixture was filtered and allowed to evaporate at room temperature until the reddish-orange crystal of 2a was obtained. The crystals were filtered and rinsed several times using ethanol, followed by drying in a desiccator. The reaction was repeated using metal salt, CuCl_2_·2H_2_O (17 mg, 0.1 mmol), to yield a complex of isomer 2b.

[Cu(ii)L(CIO_4_)](CIO_4_) (2a): Yield: 53%; m.p. 229.7–230.7 °C; UV-vis *λ* 261 nm, *ε* 6183 M^−1^ cm^−1^; *λ* 506 nm, *ε* 122 M^−1^ cm^−1^; FT-IR (KBr) (*ν*_max_/cm^−1^): 3213 (N–H), 2982 (C–H), 1670 (C=N), 1054 (C–N).

[Cu(ii)L](CIO_4_)_2_ (2b): Yield: 37%; m.p. 198.9–200.4 °C; UV-vis *λ* 259 nm, *ε* 5262 M^−1^ cm^−1^; *λ* 506 nm, *ε* 117 M^−1^ cm^−1^; FT-IR (KBr) (*ν*_max_/cm^−1^): 3205 (N–H), 2985 (C–H), 1663 (C=N), 1058 (C–N).

### 2.3. DNA binding experiments

The complexes binding studies towards the calf thymus (CT-DNA) were performed in Tris–HCI buffer (5 mM Tris, 50 mM NaCl, pH 7.1) at ambient temperature. CT-DNA absorbance at 260 nm and 280 nm were recorded, and the ratio from the absorbance value was divided to examine the purity. The 1.8–1.9 ratio indicates that CT-DNA was free from protein.^17^ In order to calculate the concentration of CT-DNA, the molar absorption coefficient of 6600 M^−1^ cm^−1^ at 260 nm was used.^18^

#### 2.3.1. Absorption spectroscopic studies

The studies on absorption titration were conducted by maintaining the copper(ii) complex concentration constant at 90 μM. Meanwhile, the concentration of CT-DNA varied from 9–135 μM. The CT-DNA addition was followed by swirling the complex-DNA solution gently for about 5 minutes to ensure it mixed well. The absorption spectra were then recorded between 200–800 nm. The data collected from the titration experiments were used to plot [DNA]/(*ε*_a_ − *ε*_f_) *versus* [DNA], and the intrinsic binding constant *K*_b_ was calculated using eqn (1):1
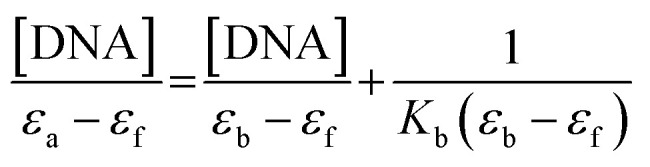
where [DNA] is the concentration of CT-DNA in base pairs, and *ε*_a_ is the apparent absorption coefficients corresponding to *A*_obsd_/[M]. *ε*_f_ and *ε*_b_ refer to the extinction coefficient for the free copper(ii) complex and the extinction coefficient for the metal(ii) complex in the fully bound form, respectively.^19^ The slope's ratio to the intercept referred to the intrinsic binding constant *K*_b_.

#### 2.3.2. Luminescence spectroscopic studies

The titration studies were carried out with a constant concentration of copper(ii) complex of 90 μM and different concentration of CT-DNA. 5 minutes was allocated to equilibrate the copper-DNA solution before the emission spectra were recorded. The emission spectra were measured at 500 nm until 550 nm, and the excitation wavelength *λ*_exc_ was set at 260 nm. The titrations stopped when the emission became constant, which indicates that the binding was saturated. The data collected from the titration experiments were used to plot *I*_0_/*I versus* [Q], and the Stern–Volmer quenching constant *K*_sv_ was determined by eqn (2):2
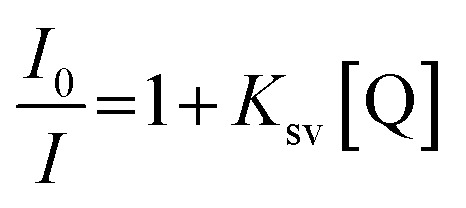
where *I* is fluorescence intensity with the quencher and *I*_0_ is the fluorescence intensity without the quencher. [*Q*] and *K*_sv_ refer to the concentration of the quencher (DNA) and Stern–Volmer quenching constant, respectively.^20^ The Stern–Volmer plot's slope referred to the Stern–Volmer quenching constant *K*_sv._

#### 2.3.3. Viscosity measurements

Ostwald viscometer was used to conduct the viscosity measurements. The viscometer was placed in a room-temperature water bath. Roughly 0.4 mM of CT-DNA solution was prepared, and the concentration was kept constant during the experiment. A digital stopwatch was used to record the time flow of the copper-DNA solution. Three readings of the flow time were recorded, and the average was calculated. The data collected was used to plot (*η*/*η*_0_)^1/3^*versus* [complex]/[DNA], where *η* is the viscosity of CT-DNA solution in the presence of complex, and *η*_0_ is the viscosity of free CT-DNA solution. The flow time of the buffer alone (*t*_0_) has to be initially measured to correct the viscosity values *η* = (*t* − t_0_)/*t*_0_.^21^

#### 2.3.4. Molecular docking

The interaction of metal complexes with DNA was analyzed using a molecular graphics program, Hex 8.0.0. CT-DNA dodecamer d(CGCGAATTCGCG)_2_ (PDB ID: 1BNA) structure was attained from the protein data bank (https://www.rcsb.org/pdb). Cif files of metal complexes were acquired from CCDC using the code (UQUSEF, BAPPUD01, CEJNMG), and then their energies were minimized using Chem3D (MM2) and saved in PDB (PROTEINDATABANK) format. The water molecules from the 1BNA were removed using Biovia Discovery Studio 2019.

### 2.4. Fabrication of sensor

**Fig. 1:**
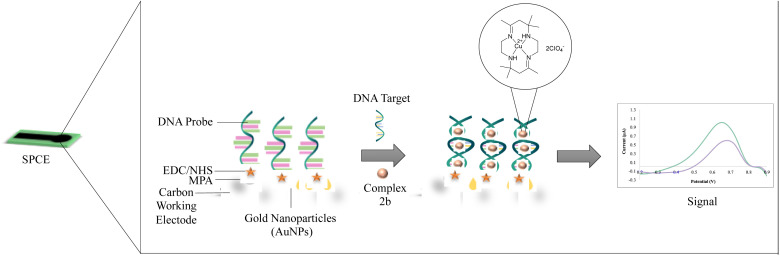
The conceptual scheme of the developed biosensor.

15 μL of gold nanoparticles, AuNPs was dropped onto screen-printed carbon electrode, SPCE and was left to react overnight. The modified electrode was then deposited with 10 μL of 3-mercaptopropionic acid, MPA (1 mM) and incubated for 3 hours in dark conditions to form covalent bonds Au–S, followed by washing with ethanol to remove excess MPA and allowed to dry. 10 μL of the coupling reagent *N*-hydroxysuccinimide:1-ethyl-3-(3-dimethylaminopropyl)carbodiimide, NHS: EDC (400 mM : 100 mM) was placed onto the modified electrode and incubated for 1 hour. The modified electrode was then rinsed with distilled water before dropping 10 μL of 5 μM probe DNA and was allowed to react overnight. The modified electrode was washed using a buffer to remove the unbound aminated probe DNA. The 10 μL mixture of 30 μM 2b complex and between 1 × 10^−13^ M to 1 × 10^−8^ M of complementary DNA was placed onto the modified electrode to allow the hybridization process for 1 hour. Then, the modified electrode was soaked in a buffer for 1 minute to remove excess complementary DNA. The DNA biosensor response was evaluated by the DPV technique. The electrochemical behaviour of the modified SPCE (AuNPs/MPA/NHS:EDC) was characterized using DPV in 0.5 mM potassium ferricyanide solution, [Fe(CN)_6_]^3−/4−^ containing 0.05 M PBS (pH 7.0) within the potential range of −0.8 V to 1.1 V at 5 mV s^−1^ scan rate. The additional DPV parameters were tabulated in Table S1 in the ESI.† The modified biosensor was determined using impedance analysis before and after the hybridization with complementary DNA (DNA target). The concept of the developed sensor is illustrated in Fig. 1.

### 2.5. Performance study of the fabricated porcine sensor

The porcine biosensor performance was studied in three categories: selectivity, linear range, reproducibility and shelf life. All the measurements were done in triplicates. The selectivity study analyzed the fabricated sensor's current intensity using different target DNA: complementary, probe, chicken and beef at 1 × 10^−8^ M DNA concentration. The linear range analysis assessed the fabricated sensor at different DNA concentrations from 1 × 10^−13^ M to 1 × 10^−8^ M. Next, the reproducibility study examined four independent sensors at complementary DNA concentrations of 1 × 10^−2^ μM and 1 × 10^−7^ μM, respectively. The shelf life experiments conducted until 23 days.

### 2.6. Real samples preparation

The new porcine DNA biosensor was tested with raw pork, chicken and beef samples. DNA from each sample was extracted according to the Spin-Column Protocol by Qiagen DNeasy Blood and Tissue Kit. The extracted DNA was hybridized with the constructed porcine DNA biosensor with the presence of copper(ii) tetraaza complex. The signal received from pork DNA was compared with chicken and beef DNA responses of the biosensor.

## 3. Results and discussion

The macrocyclic ligand [H_2_L]X_2_, X = Br (1), CIO_4_ (2) reacts with metal salts in 1 : 1 ratio in methanol solution to give copper(ii) macrocyclic complexes [Cu(ii)LX]X, X = Br (1a), CIO_4_ (2a) and [Cu(ii)L](CIO_4_)_2_ (2b) as shown in Scheme 1. The ligand and complexes were able to dissolve in common organic solvents. Spectroscopic analysis and single-crystal X-ray diffraction were used to characterize the macrocyclic ligand and copper(ii) complexes.

### 3.1. Synthesis and characterization

The synthetic yield of complex 1a and 2a are moderate at 86% and 53%, respectively. Meanwhile, complex 2b has a low yield, 37%, because the complex exists in two isomeric forms, *trans* and *cis*. The isomer isolated under slow evaporation in this study was *trans*. The *trans* isomer adopts a conformation in which the hydrogen atoms attached to the nitrogen atoms are on the opposite side of the macrocyclic plane. The *cis* isomer can be separated by recrystallization in aqueous methanol solution.^22^

The spectroscopic data of complex 1a, 2a and 2b are first discussed in this work. From the macrocyclic ligand's infrared spectrum, two bands of *ν*(N–H) were detected in the region 3466–3402 cm^−1^, corresponding to the primary amine. However, only one band of *ν*(N–H) at 3217–3205 cm^−1^ was observed for the complexes due to the secondary amine group's presence. The *ν*(N–H) stretching mode of the complexes undergoes a notable shift to lower frequencies than the free ligand. This suggests that the amino group coordinates with copper ions.^23^

The ^1^H NMR spectrum of macrocyclic ligand [H_2_L]X_2_, X = Br (1), CIO_4_ (2) showed a sharp signal *δ* 1.33–2.06, for which it was assigned to the methyl protons (–CH_3_; 9H). This signal confirmed that the cyclization between acetone and ethylene diamine has successfully occurred. The methylene protons were observed in the region *δ* 2.65–3.70 (–CH_2_; 6H). The primary amine protons were detected at *δ* 8.56 (–NH_2_; 2H) by showing a singlet signal. The ^13^C NMR spectra of the ligands showed the signal's characteristics to the imine group (C=N). All other carbons in the macrocyclic were also observed at their appropriate positions.

The absorption spectra of the macrocyclic ligand and complexes were measured in distilled water. The absorption spectra of ligands showed one intense band at 238–246 nm assignable to π–π* transition. The π–π* transition was referred to as the azomethine C=N chromophore of the ligand. A new broad peak was observed for the complexes at 504–506 nm, referring to the d–d transition. The presence of d–d transition peaks confirmed the formation of the complexes.

The X-ray molecular structures of complexes 1a, 2a and 2b have been reported earlier by other literature using different reactants and methods.^24–27^ Complex 1a has monoclinic crystal system with space group *P*21/*c*, *a* = 17.8403(16) Å, *b* = 15.4797(13) Å, *c* = 17.2018(14) Å, *β* = 112.037(2)°, *Z* = 12 and *V* = 4403.4(7) Å^3^. The tetraaza ligand coordinated to the copper atom through all four nitrogen atoms. One bromo atom was also coordinated to the metal centre copper. Hence, the complex possesses square pyramidal geometry. Another bromine ion acts as a counter anion of the monocation complex.

Complex 2a has monoclinic crystal system with space group *P*21/*c*, *a* = 10.4803(11) Å, *b* = 16.9627(17) Å, *c* = 13.8527(15) Å, *β* = 105.145(3)°, *Z* = 8 and *V* = 2377.1(4) Å^3^. The copper metal centre and one perchlorate ion were coordinated to all four nitrogen atoms, making the complex possess square pyramidal geometry. The other perchlorate anion stays as the counter anion of the complex.

For the complex 2b, the crystal system is monoclinic with the space group *P*21/*c*, *a* = 10.3221(7) Å, *b* = 10.6267(7) Å, *c* = 11.0213(7) Å, *β* = 111.8020(18)°, *Z* = 4 and *V* = 1122.45(13) Å^3^. Only the copper metal centre coordinated to all four nitrogen atoms. Hence, the complex possesses a square planar geometry. The two perchlorate anions balance the complex by having a +2 charge.

### 3.2. DNA binding studies

#### 3.2.1. Absorption spectra

Electronic absorption was among the most critical methods for studying metal complexes' DNA binding. Intercalation binding mode exhibits significant hypochromism and red shift due to the sharing of pi–pi orbital between complexes and DNA base pairs. Meanwhile, the non-intercalative or electrostatic mode is correlated with hyperchromism or hypochromism with slight or no shift. The absorption spectra of 1a, 2a and 2b complexes in the absence and presence of CT-DNA are depicted in ESI 1.† The addition of CT-DNA contributed to the increase in the concentration of CT-DNA. This result leads to a slight decrease in the absorption peaks (hypochromism) at 257 nm, 261 nm and 259 nm, with *H*% = 100(*A*_free_ − *A*_bound_)/*A*_free_ being 3%, 5% and 6% and a slight shift of 1 nm red shift, 1 nm blue shift and 1 nm redshift, respectively (Table 1).

**Table 1:** The results from the absorption and luminescence binding study for each complex

Complex	Absorption Study			Luminescence study	
	Hypochromism (%)	Red or blue shift (nm)	Binding constant *K*_b_ (M^−1^)	Fluorescence decreasing factor	Stern volmer quenching constant, *K*_sv_ (M^−1^)
1a	3	1	2.24 × 10^4^	0.85	2.74 × 10^4^
2a	5	1	1.53 × 10^4^	0.79	2.54 × 10^4^
2b	6	1	5.02 × 10^4^	0.84	4.01 × 10^4^

The small hypochromism and slight red or blueshift proposed that the complexes interact electrostatically with DNA. Electrostatic interactions involve the external binding between positively charged complexes with negatively charged DNA phosphates. The changes in spectral characteristics observed were in agreement with the tetraaza copper complex, [CuL^1^]^2+^, where L^1^ = 1,4,8,11-tetraazacyclotetradecane and hexaaza copper complex [CuL^2^]^2+^, where L^2^ = 3,10-bis(2-hydroxyethyl)-1,3,5,8,10,12-hexaazacyclotetradecane that are reported to bind electrostatically with DNA.^28^

The DNA binding strength can be determined from the intrinsic binding constant *K*_b_ for 1a, 2a and 2b complexes were 2.24 × 10^4^ M^−1^, 1.53 × 10^4^ M^−1^ and 5.02 × 10^4^ M^−1^, respectively. For comparison, the complexes that interact with DNA electrostatically have intrinsic binding constant *K*_b_ in the range of 2.5 × 10^4^ M^−1^ to 8.5 × 10^4^ M^−1^.^29–33^ These complexes' binding strengths at magnitude 10^4^ are identical to complexes 1a, 2a, and 2b. Hence, the complexes clearly bind to DNA by electrostatic mode. The complexes' binding strengths were arranged as followed 2b > 1a > 2a. The differences in the binding strength might be because of the dissimilar charge and counter ion of metal complexes. It can be explained by Coulomb's law which states that the electrostatic attraction force is directly proportional to the magnitude of positively and negatively charged ions and inversely proportional to the square value of the distance between the ionic charge. For complex 2b, the complex carries a +2 charge, thus making the strength of the electrostatic attraction between the complex and DNA higher than the other complexes. Meanwhile, for 1a and 2a, both complexes carry +1 charges. However, complex 1a has higher binding strength than complex 2a due to the smaller size of the monoatomic bromide anion ligand compared to the polyatomic perchlorate anion ligand. The small size of the anion ligand allowed the complex to interact more strongly with the DNA.

#### 3.2.2. Luminescence spectra

Luminescence spectroscopy is frequently utilized in the interaction studies between complexes and DNA due to its sensitivity. This technique provided information about the changes happening in the microenvironment of DNA upon complex binding. Emission peaks at 522 nm were observed for all three complexes 1a, 2a and 2b by dissolving them in Tris–HCl buffer. Emission intensities of complexes 1a, 2a, and 2b decreased upon the addition of CT-DNA by a factor of 0.85, 0.79 and 0.84, respectively, as shown in ESI 2.† The differences in the fluorescence emission intensities indicate that macrocyclic complexes' interaction with DNA changed the fluorophore microenvironment. The fluorescence emission intensities of the complexes decreased after interaction with DNA. These might be due to the masking or burial of complexes fluorophores when interacting between the DNA base stacking. Besides that, the photoelectron transfer from DNA bases to the MLCT excites the complex state. The spectra that showed only a slight decrease in the emission intensities for complexes 1a, 2a and 2b were found to be similar to the ruthenium(ii) complex, *trans*-[Ru(Hmel)_2_(dmso)_2_] (Hmel = meloxicam), which happened to interact with DNA by electrostatic.^34^ In addition, the organotin(iv) complex that binds electrostatically to DNA also demonstrated minimal intensity changes when DNA is added.^35^

The Stern Volmer quenching constant was applied to analyse synthesised complexes' quenching strength. The value of the quenching constant for 1a, 2a and 2b complexes were 2.74 × 10^4^ M^−1^, 2.54 × 10^4^ M^−1^ and 4.01 × 10^4^ M^−1^, respectively, as illustrated in Table 1. Complex 2b had the highest *K*_sv_ value. It can be concluded that the quenching ability of complex 2b was the strongest compared to other complexes. The complex's higher quenching ability implies a higher DNA binding ability towards the complex. In this case, complex 2b favourably interacts with DNA compared to complex 1a and 2a. The magnitude of Stern Volmer quenching constant for complexes 1a, 2a and 2b were similar to the non-intercalator compounds, which interact electrostatically with DNA.^30,32,36,37^

#### 3.2.3. Viscosity measurements

Hydrodynamic measurements are responsive to changes in length, and among the tests used are viscosity and sedimentation tests. When dealing with non-crystallographic results, it was the least uncertain and most worthy binding test in solutions.^38,39^ The lengthening of the DNA helix is often associated with classical intercalation due to the ligand's intercalation between the DNA base pairs lengthening the DNA helix. Thus, it leads to an increase in the viscosity of DNA. In contrast, partial intercalation caused the DNA helix to bend or kink and consequently reduced its effective length, implying a decrease in DNA viscosity. Meanwhile, groove binding and electrostatic interactions result in minimal or no DNA viscosity changes.^40,41^

**Fig. 2:**
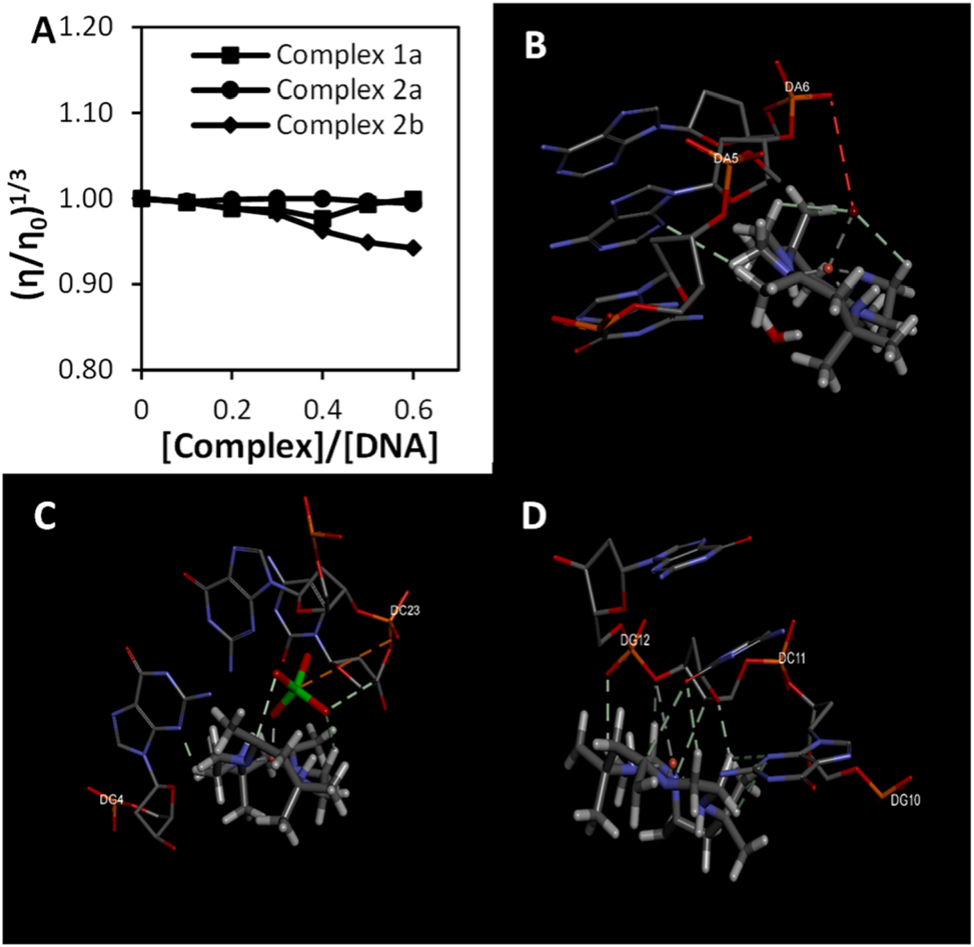
The binding study evaluations using viscosity and computational study (a) effects of the increasing amount of 1a, 2a, and 2b complexes on the relative viscosity of CT-DNA (b–d) molecular docked structures of the complex with DNA dodecamer duplex of sequence d(CGCGAATTCGCG)_2_ (PDB 1D: 1BNA). (b) Complex 1a, (c) complex 2a, (d) complex 2b.

Fig. 2(a) illustrates the changes in DNA relative viscosity after adding complexes 1a, 2a, and 2b. As the complexes' concentration increased, DNA solutions' viscosity displayed no significant changes, less than 0.05 (*η*/*η*_0_)^1/3^. The findings indicated that complexes 1a, 2a, and 2b interact with DNA electrostatically. These behaviours were similar to the [Ru(bpy)_3_]^2+^ (bpy = bipyridine), a known complex that has an electrostatic binding mode when interacting with DNA. Notably, complex 2b interacts strongly with DNA compared to complexes 1a and 2a because the extent of DNA viscosity changes was the biggest. These results and the spectroscopic results mentioned above were consistent with each other.

#### 3.2.4. Molecular docking

Molecular docking is a promising technique that can help design drugs and mechanical studies between small molecules and DNA binding sites. The docking studies have been conducted on complexes 1a, 2a and 2b with B-DNA (PDB ID: 1BNA) to explore a possible binding site, mode of interaction and binding affinity. Fig. 2(b–d) shows that the complexes bind electrostatically toward the DNA groove. The relative binding energies of the docked complex 1a, 2a, and 2b were −223.88, −221.94 and −224.35, respectively. Stronger DNA binding affinity was demonstrated by having more negative binding energy. Thus, the DNA binding affinities can be arranged as follows 2b > 1a > 2a, consistent with the results of the electronic absorption titration, luminescence titration and viscosity measurements. Furthermore, the interaction of complexes and DNA were illustrated to have certain hydrogen bonding.

### 3.3. Electrochemical behaviour of modified SPCE in [Fe(CN)_6_]^3−/4−^

**Fig. 3:**
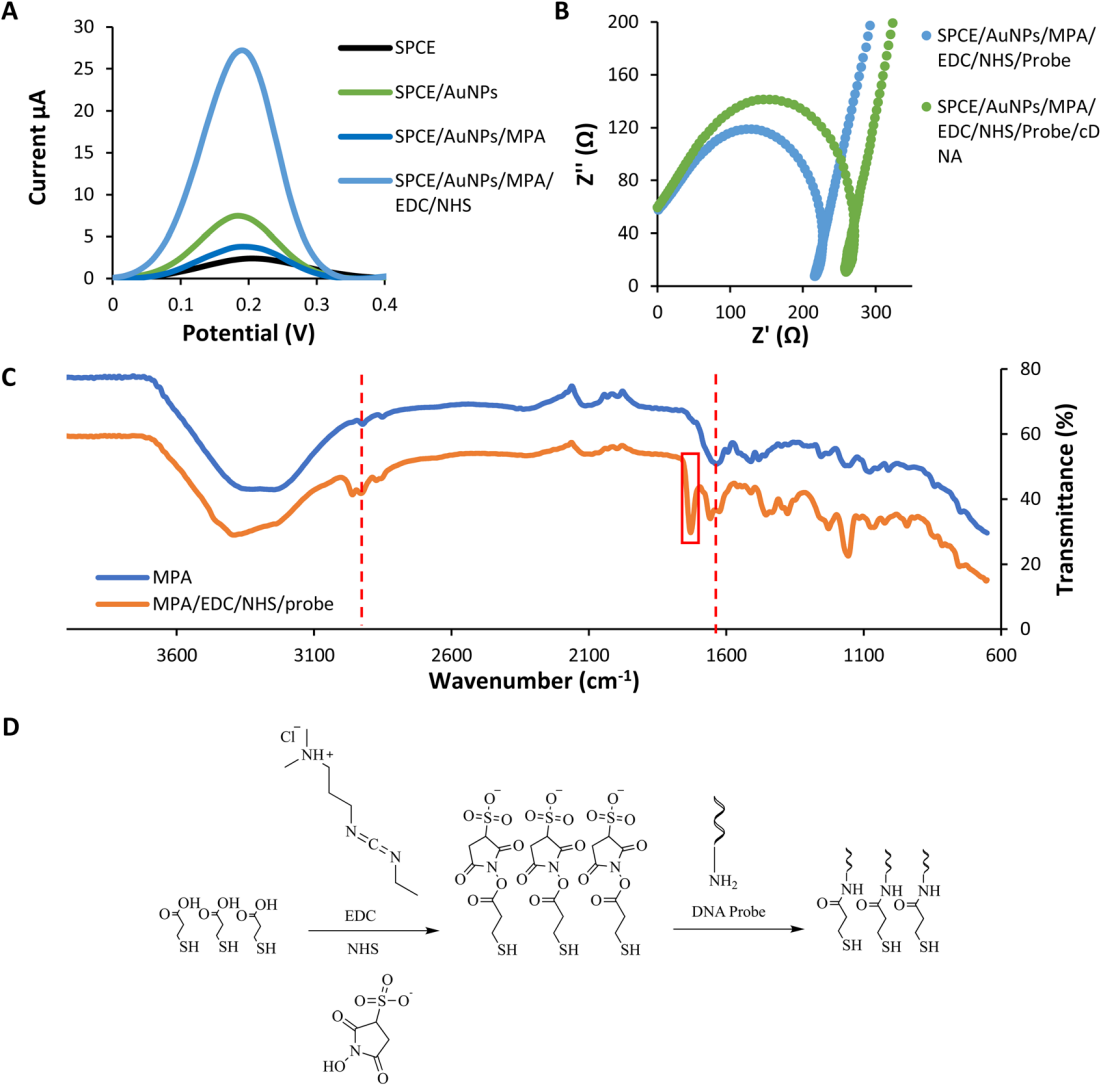
Layers analysis of the modified SPCE (a) the differential pulse voltammogram response of each self-assembled monolayer (b) the impedance spectroscopy of before and after DNA hybridization in potassium ferricyanide solution (Fe(CN)_6_^3−/4−^) 0.5 mM at 5 mV s^−1^ scan rate (c) the infrared spectrum of the modified biosensor (d) the reaction scheme of MPA/EDC/NHS/Probe.

The layer of modification on SPCE was analyzed using DPV in potassium ferricyanide solution (Fe(CN)_6_^3−/4−^) 0.5 mM within the range of −0.8 V to 1.1 V at a 5 mV s^−1^ scan rate (Fig. 3(a)). This characterization was conducted to study the electron transfer between each layer of the DNA biosensor and the electrode.

The immobilization of the gold nanoparticles (AuNPs) enhances the current peak compared to the bare SPCE due to the gold's conductive properties.^42^ Gold nanoparticles have high electron transfer performance on the modified electrode. In contrast, the peak later decreases dramatically after the assembling of MPA onto the gold layer. It is because of the interruption of electron transfer caused by the MPA layers.^42^ MPA formed a dense layer on the gold surface and could be ascribed to the strong chain between MPA. The immobilization of EDC–NHS induces the current peak higher compared to the previous layer. The surface of –COOH groups of MPA are activated by EDC and converted into active esters intermediates. NHS converts the intermediates of EDC into a more stable active ester. This intermediate product showed good electrochemical activity.^43^ According to Trammell *et al.*, the intermediate product could delocalize electrons, thus enhancing the electron tunnelling and electrons' movement.^44^ All these data indicated that the fabrication of each assembled monolayer was successful.

Besides DPV analysis, the characterization of every single layer was analyzed using the impedance technique (Fig. 3(b)). In impedance analysis, we are focusing on before and after DNA hybridization. Based on the Nyquist plot, the resistance charge transfer value (*R*_CT_) increased due to increased negative charge from the DNA backbone after the hybridization process.^45^ The negative charge repelling between DNA and potassium ferricyanide solution cause a DNA blocking effect. This effect prevents electron transfer through the electrode surface.^46^ The R_CT_ value increased due to the DNA blocking effect.^47,48^

### 3.4. Layers analysis using infrared

From the infrared analysis (Fig. 3(c and d)), the assembly of the MPA layer was confirmed by the appearance of medium C–H stretching and strong C=O acid stretching at 2923 cm^−1^ and 1635 cm^−1^. The successful functionalization of the DNA probe onto the layer was determined by the presence of a new vibrational peak of C=O amide at 1729 cm^−1^ corresponds to the formation of a covalent bond between MPA and the aminated probe.^49^ The functionalization happens with the use of coupling agent EDC and NHS. Another important vibrational peak indicating the DNA probe's functionalization was the strong C=O amide stretching of the oligonucleotides at 1658 cm^−1^.^50^

### 3.5. Analytical performance

The designed biosensor was fabricated based on an MPA self-assembly monolayer formation on the SPCE, modified with AuNPs. The MPA molecule consists of a thiol functional group with a high affinity toward gold. In contrast, the carboxyl group at the other end of the MPA molecule is suitable for covalently attached to DNA after the activation process of the MPA. Activation procedures involved the use of EDC and also NHS. Table S2 in ESI† shows all of the parameters optimized for fabricating a porcine DNA biosensor with slight modification from the previous studies.^51^

**Fig. 4:**
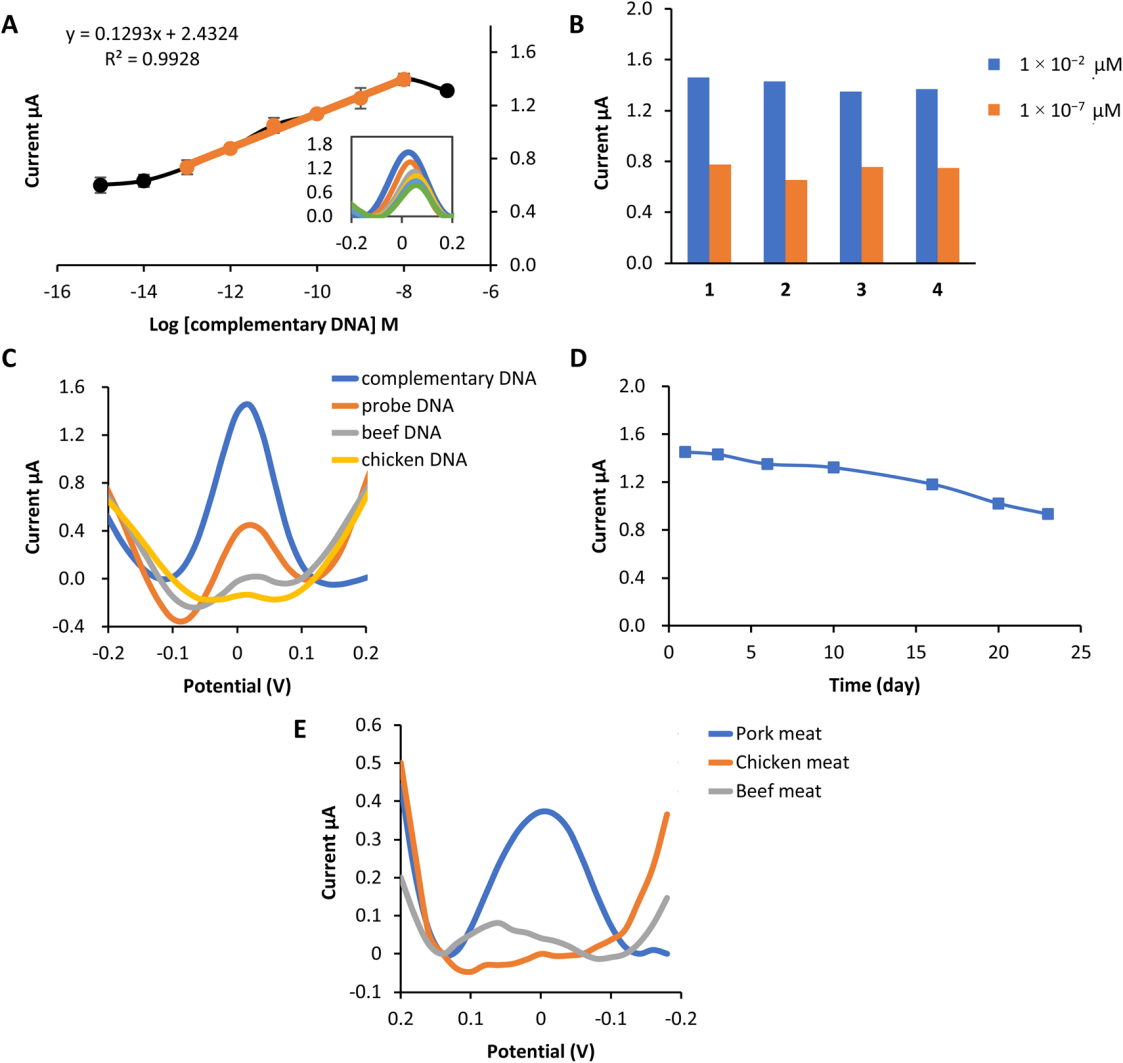
Performance of the biosensor and real sample analysis (a) the linear response range between 1 × 10^−13^ M–1 × 10^−8^ M and the dynamic range of the porcine DNA biosensor. (b) Reproducibility of the porcine DNA biosensor at concentrations of 1 × 10^−2^ μM and 1 × 10^−7^ μM. (c) Selectivity of the porcine DNA biosensor based on complex 2b as redox indicator toward the determination of probe DNA only, complementary DNA, beef DNA and chicken DNA at 1 × 10^−2^ μM. (d) Shelf life of the porcine DNA biosensor at the concentration of 1 × 10^−2^ μM store at 4 °C (e) A comparative analysis of the response of the constructed porcine DNA biosensor with extracted DNA from pork, chicken and beef meat.

This biosensor's linear concentration range based on the complex 2b redox label was 1 × 10^−13^ M–1 × 10^−8^ M with the correlation coefficient, *R*^2^ = 0.993 [Fig. 4(a)]. Within this range, the current response increases correspondingly, proving that more hybridization occurs as the concentration of target DNA increases. This electrochemical biosensor's detection limit (LOD) was 5.7 × 10^−14^ M calculated using LOD = [(mean blank + 3 standard deviation) − intercept]/slope.

A reproducibility analysis was carried out using two different concentrations to assess the reusability of this electrochemical biosensor. Fig. 4(b) proved that the porcine DNA biosensor was able to provide a repeatable DPV response of 1 × 10^−2^ μM and 1 × 10^−7^ μM. The average reproducibility relative standard deviation (RSD) of each calibration point evaluated using this new DNA biosensor was estimated to be about 3.7% (*n* = 4) and 7.3% (*n* = 4), respectively.

The selectivity study of the DNA biosensor for detecting porcine DNA using copper complex 2b as the redox indicator is presented in Fig. 4(c). A selectivity study was conducted by comparing the DPV response of the DNA biosensor toward DNA probe only, complementary DNA (cDNA), beef DNA, and chicken DNA at 1 × 10^−8^ M (concentration). Hybridization of probe DNA showed to be profoundly specific toward complementary DNA of porcine (cDNA) as the hybridization response of complex 2b gave the highest current compared to other DNA. Peak current response at 1.45 μA indicates that complex 2b had been successfully interacting electrostatically with the phosphate backbone of immobilized probe DNA and cDNA. A significant difference can be seen with the immobilized single-stranded DNA probe before and after hybridization with other DNA. The DPV response of probe DNA only is (0.45 μA), while beef DNA and chicken DNA showed almost no biosensor response. This might be due to the ability of complex 2b to bind to the phosphate group of probe DNA only. Meanwhile, no complex 2b interacts with probe DNA after hybridization with other DNA. This is because complex 2b and the single-stranded chicken and beef are mixed first before the hybridization process, so complex 2b can bind to the DNA phosphate backbone before being deposited onto the modified electrode. However, they are washed off together because of the inability to form duplex DNA with the porcine DNA probes, hence producing practically no response.^52^

The lifetime of the biosensor is demonstrated in Fig. 4(d). From the study, the biosensor response remained stable from day 1 to day 16, in which the current was reduced to 81% of its initial value after being kept at 4 °C. Starting from day 20, the biosensor response decreased to 70% and below. This is due to the degradation of the biosensor system, such as gold nanoparticles (AuNPs), MPA or probe DNA from the electrode surface. Therefore, reduced the amount of probe subjected to target complementary DNA, eventually leading to the decrease in biosensor signal.

### 3.6. Porcine detection

The effectiveness of the constructed porcine DNA biosensor was tested with several kinds of meat. Pork, chicken and beef meat were used in this experiment. DNA from each sample was extracted using Qiagen DNeasy Blood and Tissue Kit. It is obviously shown that the DNA signal from the extracted pork meat gives the highest current value compared to chicken meat and beef meat (Fig. 4(e)). Chicken and beef meat samples give almost no response to the biosensor. This newly porcine DNA biosensor has excellent selectivity towards pork meat extracted DNA.

### 3.7. Comparison on the performance of the reported electrochemical porcine biosensor

The comparison of the constructed biosensor performances with previously reported electrochemical porcine DNA biosensors using different DNA matrix immobilization and redox compounds is tabulated in Table 2. This comparison is essential in order to validate the developed biosensor. From the table, the limit of detection obtained in this study is the lowest and has a broad linear range. Thus, we concluded the high sensitivity of the constructed biosensor compared to other DNA ESI† and redox compounds due to the large surface area of AuNPs, which have increased the DNA binding capacity. Another essential factor that enhances the sensitivity of the constructed biosensor is the four amine groups contained in the copper(ii) tetraaza complex redox compound. This compound has a very high interaction with DNA and can increase the DNA binding capacity.

**Table 2:** Comparison on the performance of developed and reported porcine DNA biosensors

Matrix immobilization	Redox compound	Linear range	LOD	References
SPCE/AuNPs/MPA/EDC–NHS	Copper(ii) tetraaza complex	1 × 10^−13^ M– 1 × 10^−8^ M	5.7 × 10^−14^ M	This work
SPCE/AuNPs/NBA-NAS	Ruthenium(ii) complex	1 × 10^−13^ M–1 × 10^−8^ M	—	53
Screen printed carbon–reduced graphene oxide (SPC–RGO) electrode	Guanine	0–10 μg mL^−1^	1.8 × 10^−6^ g mL^−1^	54
SPCE/AuNPs	Methylene blue	0.1–5.0 μg mL^−1^	5.8 × 10^−7^ g mL^−1^	55
SPCE	Luminol	—	1.0 × 10^−10^ g mL^−1^	56
Graphene biochips	Ruthenium hexamine complex	—	1.0 × 10^−7^ g mL^−1^	57
Disposable electrochemical printed (dep) chips	Hoechst33258	—	2.0 × 10^−5^ g mL^−1^	58
SPCE/AuNPs/SiNSs/GA	Hexaferrocenium complex	1 × 10^−12^ to 1 × 10^−9^ M	4.8 × 10^−14^ M	59

## 4. Conclusions

In summary, the interactions between tetraaza macrocyclic complexes [Cu(ii)LBr]Br (1a), [Cu(ii)L(CIO_4_)](CIO_4_) (2a) and [Cu(ii)L](CIO_4_)_2_ (2b) where L = 5,5,7,12,12,14-hexamethyl-1,4,8,11-tetraazacyclotetradeca-7,14-diene, with DNA were studied by spectroscopic techniques and viscosity measurements. The results collected proved that these complexes interact with DNA by electrostatic mode. The strength of binding constants, *K*_b_ of complexes, were arranged in 2b > 1a > 2a. A sensitive and selective electrochemical porcine biosensor using copper(ii) tetraaza complexes 2b has been established. The developed sensor exhibits a broad linear range and good repeatability. The real sample analysis indicated that the biosensor has excellent selectivity to the extracted pork DNA. These results displayed that the copper(ii) complex 2b has the potential to be used as an indicator in the prototype development of a porcine detection biosensor.

## Author contributions

Noraisyah Abdul Kadir Jilani: writing-original draft, formal analysis, data curation, methodology, validation, investigation. Emma Izzati Zakariah: writing-original draft, data curation. Eda Yuhana Ariffin: writing-original draft, data curation, validation, investigation. Suhaila Sapari: writing-original draft. Devika Nokarajoo: formal analysis, investigation. Bohari Yamin: supervision, resources. Siti Aishah Hasbullah: writing-review & editing, supervision, resources.

## Conflicts of interest

There are no conflicts to declare.

## Supplementary Material

RA-013-D2RA05701H-s001
