# Supplementary material for: Highly sensitive pork meat detection using copper(ii) tetraaza complex by electrochemical biosensor
Source: RSC Adv. 2023 Jan 11;13(3):2104–14. doi: 10.1039/d2ra05701h (PMC9832347; doi:10.1039/d2ra05701h)
Supplement: RA-013-D2RA05701H-s001 [file RA-013-D2RA05701H-s001.pdf]

This journal is © The Royal Society of Chemistry 2022

Supplementary materials

Highly sensitive pork meat detection using copper(II) tetraaza complex by electrochemical biosensor

Noraisyah Abdul Kadir Jilani, Emma Izzati Zakariah, Eda Yuhana-Ariffin, Suhaila Sapari, Devika Nokarajoo, Bohari Yamin, Siti Aishah Hasbullah*

Contents

1. Absorption spectra DNA titration S1
2. Luminescence spectra DNA titration S2
3. Optimized DPV parameters for porcine detection S2
4. Porcine DNA biosensor parameter optimization S2

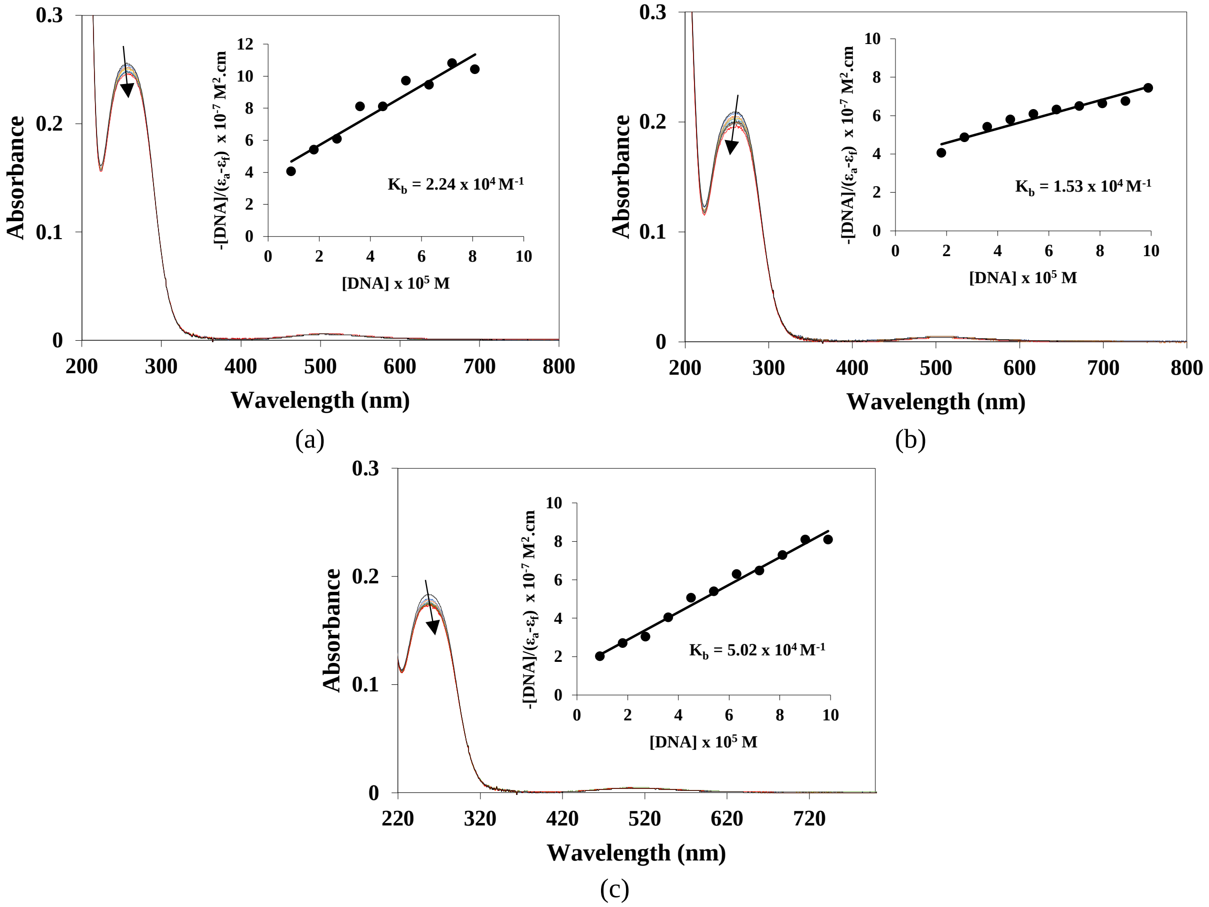

Fig. S1. Absorption spectra for complex (a) **1a**, (b) **2a**, (c) **2b** in Tris-HCl buffer after DNA addition. [**1a, 2a, 2b**] = 90 μM, [DNA] = 0–135 μM. Inset displayed reciprocal plot [DNA]/(ε_a-ε_f) vs. [DNA] for DNA titration with **1a, 2a, 2b**.

.

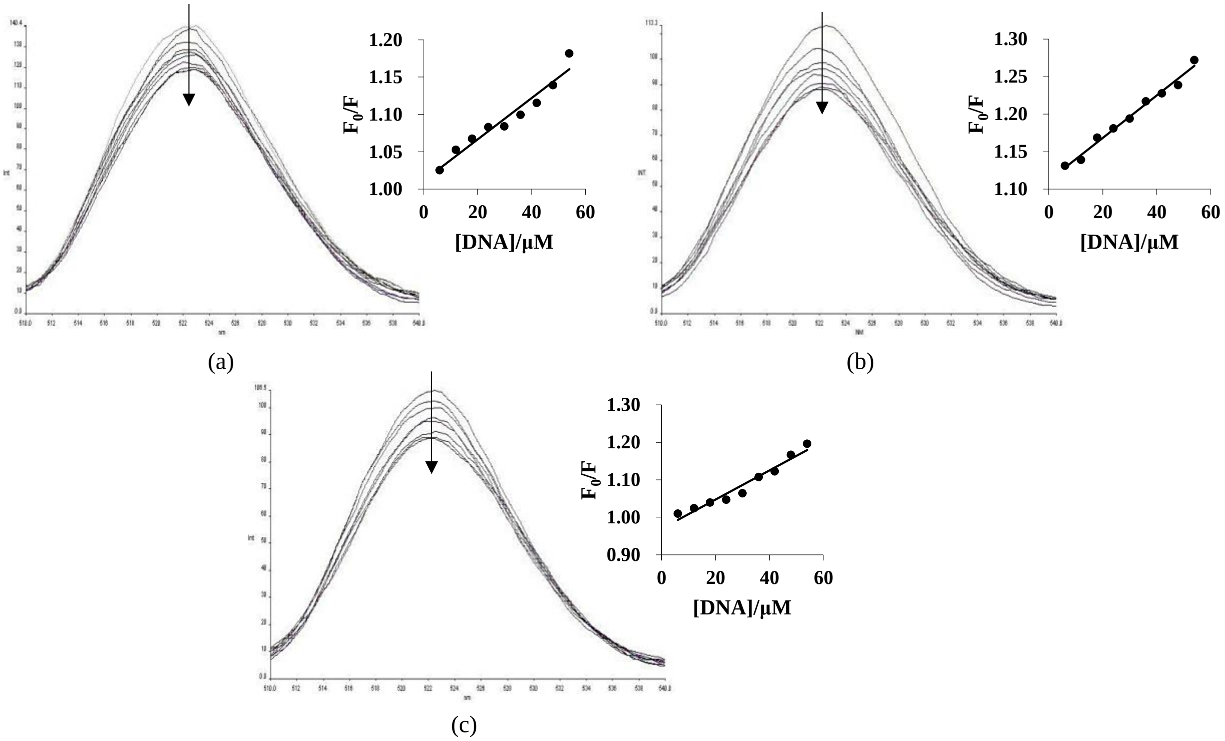

Fig. S2. Fluorescence emission spectrum of complex (a) **1a**, (b) **2a**, (c) **2b** as the volume of CT-DNA increases. The inset demonstrates the Stern Volmer plot for the binding with CT-DNA

Table S1. Optimized DPV parameters for porcine detection

**Parameters**	**Results**
Initial potential (V)	-0.8
Final potential (V)	1.1
Pulse amplitude (mV)	20
Pulse time (ms)	80
Scan rate (mVs^{-1})	5
Equilibration time (s)	5

Table S2. Porcine DNA biosensor parameter optimization

**Parameters**	**Optimum amount**
Amount of AuNPs	15 µL
Concentration of MPA	1 mM
Concentration of NHS: EDC	100 mM:400mM
Concentration of potassium phosphate buffer	0.05 M
pH of Potassium phosphate buffer	pH 7
Concentration of DNA probe	5 µM
Hybridization time	1 hour
Concentration of complex **2b**	3×10^{-5} M
